# Seeing Inscriptions on the Shroud of Turin: The Role of Psychological Influences in the Perception of Writing

**DOI:** 10.1371/journal.pone.0136860

**Published:** 2015-10-28

**Authors:** Timothy R. Jordan, Mercedes Sheen, Lily Abedipour, Kevin B. Paterson

**Affiliations:** 1 Department of Psychology, Zayed University, Dubai, UAE; 2 School of Psychology, University of Leicester, Leicester, United Kingdom; University of Amsterdam, NETHERLANDS

## Abstract

The Shroud of Turin (hereafter *the Shroud*) is one of the most widely known and widely studied artifacts in existence, with enormous historical and religious significance. For years, the Shroud has inspired worldwide interest in images on its fabric which appear to be of the body and face of a man executed in a manner consistent with crucifixion, and many believe that these images were formed in the Shroud’s fibers during the Resurrection of Jesus of Nazareth. But, more recently, other reports have suggested that the Shroud also contains evidence of inscriptions, and these reports have been used to add crucial support to the view that the Shroud is the burial cloth of Jesus. Unfortunately, these reports of inscriptions are based on marks that are barely visible on the Shroud, even when images are enhanced, and the actual existence of writing on the Shroud is still a matter of considerable debate. Here we discuss previous evidence concerning the psychological processes involved generally in the perception of writing, and especially when letters and words are indistinct. We then report two experiments in which the influence of religious context on perception of inscriptions was addressed specifically, using an image of woven fabric (modern linen) containing no writing and with no religious provenance. This image was viewed in two different contexts: in the Religious Context, participants were informed that the image was of a linen artifact that was important to the Christian faith whereas, in the non-religious Neutral Context, participants were informed that the image was of a simple piece of linen. Both groups were told that the image may contain faint words and were asked to report any words they could see. All participants detected words on the image, and indicated that these words were visible and were able to trace on the image the words they detected. In each experiment, more religious words were detected in the Religious Context condition than in the Neutral Context condition whereas the two contexts showed no effect on the number of non-religious words detected, indicating that religious context had a specific effect on the perception of illusory writing. Indeed, in the Neutral Context condition, no religious words at all were reported in either experiment. These findings suggest that images of woven material, like linen, inspire illusory perceptions of writing and that the nature of these perceptions is influenced considerably by the religious expectations of observers. As a consequence, the normal psychological processes underlying perception of writing, and the tendency of these processes to produce illusory perceptions, should be an essential consideration when addressing the existence of religious inscriptions on religious artifacts such as the Shroud of Turin.

## Introduction

The Shroud of Turin (hereafter *the Shroud*) is one of the most widely known and widely studied artifacts in existence, with enormous historical and religious significance (e.g., [1–5]). However, the exact provenance of the Shroud is unclear. On the one hand, some argue that the Shroud is over 2000 years old while others have argued that the Shroud is from medieval times, dating from the period 1260–1390 (e.g., [6–12]). Nevertheless, there is general agreement that the Shroud bears images of the body and face of a man who appears to have been executed in a manner consistent with crucifixion, and many believe the Shroud is actually the burial cloth in which Jesus of Nazareth was wrapped before being placed in a tomb about 2000 years ago (see [13]). Indeed, some researchers (e.g., [14, 10]) have even suggested that the image was formed by exposure to a brief, intense source of energy coming from a body wrapped in the Shroud, and that the nature of the image within the fabric of the Shroud is consistent with such an event.

But detecting images on the Shroud is not an easy task, and this issue has been acknowledged repeatedly in the literature (for a review, see [15]). For example, the famous images of the body and face are barely visible without using photographic and image-processing techniques, and these techniques have raised considerable concerns about the nature and validity of the images they produce (e.g. [16]). In addition, the Shroud has accumulated centuries’ of grime and innumerable marks and impressions due to effects of fire, water, and creasing, all of which add considerable complications to the task of detecting images in the fabric that can actually help determine the Shroud’s true provenance.

### The Detection of Inscriptions on the Shroud

Although the images long associated with the Shroud are those of a body and face, relatively recent claims concerning the existence of inscriptions have the potential to link the Shroud precisely to the crucifixion of Jesus of Nazareth. Since the 1980s, several studies have suggested that images of the Shroud contain evidence of legible inscriptions and that these inscriptions provide crucial written information concerning the history of the Shroud and its religious significance (e.g., [17–20]). In particular, researchers have claimed to have discovered inscriptions written in Latin, Greek, and Aramaic which are of extremely low contrast (and invisible to the naked eye) but which were revealed after photographic images of the Shroud were digitally processed. Moreover, many of these inscriptions appear to be consistent with the view that the Shroud is the burial cloth of Jesus of Nazareth. For example, Marastoni identified, among others, the inscription “NAZARE” (which may stand for “Nazarenus”) and “IN NECE” (“at death”) on the side of the face. Perhaps most fascinating is the claim by Barbara Frale that she has discovered writing on the Shroud that comes from the burial certificate of Jesus of Nazareth, and which transferred from the original document when it was fixed to the Shroud for purposes of identification [20]. Clearly, the existence of such writing on the Shroud would have immense relevance and importance for determining its actual provenance.

### The Visual Bases for Detecting Inscriptions on the Shroud

The notion that inscriptions are present on the Shroud has inspired considerable debate, and critics have focused on the nature of the photographic images and digital processes that have been used to provide evidence of writing (for reviews, see [15, 16]). Of particular concern is that the visual content of the images of the Shroud that are used by researchers is very indistinct and depends heavily on the nature of the photography and image manipulation that has been employed. For example, in the original study of the Shroud by Enrie [21], the photographs taken used orthochromatic film which records a quasi-binary image in black and white without mid-tone greys, and so much of the visual information present on the Shroud itself was altered (and even discarded) by this process. Under these conditions, enhancing the contrast of the original images of the Shroud is likely to produce shading and contours that resemble letter strokes, letters, and even complete words, but which are not actually present on the artifact itself. Similar problems are also present with more recent photographs of the Shroud where researchers have attempted to enhance these images in order to reveal visual content that is so faded or of such low contrast that it is normally invisible (e.g., see [16, 22]). Consequently, when inscriptions are detected in images of the Shroud, it is difficult to be certain that the visual content giving rise to the perception of writing actually exists on the fabric itself.

### The Illusory Perception of Writing

But while the nature of photographic images and image enhancement raises concerns over the existence of inscriptions on the Shroud, a crucial component of identifying writing on the Shroud that has received much less attention is the way in which written language is normally processed by human observers. The major problem this normal processing poses for the detection of *genuine* inscriptions is that the visual conditions provided by images of the Shroud, either normal or digitally enhanced, provide indistinct sensory input in which the perception of letters and words is likely to occur even when no writing actually exists. Indeed, it is well established that word recognition relies on many different visual components (e.g., [23–29]) and so it may well be the case that many different low-level visual properties contribute to the illusory perception of words when images of the Shroud are scrutinized for evidence of writing. Moreover, it is well known that illusory perceptions generally can be experienced by humans when the physical information that normally produces these perceptions is not actually present. For example, when looking at such things as lunar craters, arbitrary wisps of cloud, or even toasted food, humans often have the impression of seeing a face even though the visual content of the image has no actual link to the presence of a face (e.g., [30–36]). These illusions seem to reflect a human predisposition to impose structure on sensory input, with the effect that the reported detection of faces can occur even when no facial image exists. But while the illusory perception of faces (and other objects) is widely-reported, it is less widely known that similar events can also occur for the perception of letters and words.

An indication of illusory letter perception is provided by Rieth, Lee, Lui, Tian and Huber [37] who showed observers single letters embedded in various levels of random visual noise (essentially, patterns of randomly-selected light and dark pixels) and asked them to report if a letter was present on each occasion. Using a technique called “reverse correlation” [38], Rieth et al then computed correlations between the images that were actually presented and the detection of letters made by observers, and found that observers detected letters even when images consisted only of pure visual noise. Moreover, when this illusory letter detection occurred, greater fMRI activation was observed in several cortical regions, including the precuneus, an area generally involved in top-down processing of stimuli, and the left superior parietal lobule, an area associated with the processing of real letter and real word stimuli (see also [30, 39]). Consequently, it seems not only that pure visual noise can trigger the illusory detection of letters but also that, when illusory letters are detected, the brain activations produced resemble those elicited when real letters and real words are processed.

These findings have considerable implications for the detection of writing on the Shroud. In particular, because of the indistinct contours and visually-noisy environment provided generally by images of the Shroud (either normal or enhanced), observers may often detect letters and words in these images when no evidence produced by the existence of letters or words is actually present. Indeed, other evidence of the compelling nature of illusory letter perception comes from a study by Jordan, Thomas and Scott-Brown [40]. Here, the two exterior letters of four-letter English words (e.g., h and d from hand) were shown to observers as they would appear in real words (h d), and only noise characters composed of diagonal lines were presented in the two interior positions. When the real letters in each stimulus were made barely visible (by altering viewing distance), observers readily saw a range of illusory contours where the noise characters were located, such that each stimulus looked like an entire four-letter word (e.g., hand) with all four letters present in their appropriate locations (the *Illusory Letters Phenomenon*). Indeed, the effect was so compelling that participants believed that whole words were actually presented on each occasion and were able to trace the outline of four letters in each stimulus, including letters in interior positions where no letters were present. More recently, the Illusory Letters Phenomenon has also been observed for Arabic stimuli [41], indicating that the illusory perception of letters to form whole words is not restricted to Latinate languages. As Jordan and colleagues have argued, it seems likely that powerful top-down resources can combine with low-contrast, irrelevant, or incomplete visual input to produce illusory perceptions of complete letters and complete words, and reading generally may occur as a process that is augmented by illusory percepts. Indeed, these effects may be even greater when illusory letters and words are detected in the context of whole phrases and sentences. In particular, when words in printed sentences are masked by visual noise, observers are more likely to identify words that are consistent with information that can be extracted from the remainder of the sentence (e.g., [42–44]). Thus, when identifying inscriptions on the Shroud, the perceived identities of illusory letters and words may be influenced by similar processing of other parts of the image, and this interactive processing may inspire the illusory detection of coherent phrases and sentences that some researchers have claimed are present on the Shroud (e.g., [17–20]).

So, somewhat paradoxically, the task of identifying genuine writing on the Shroud is likely to be hampered by processes that are normally highly effective for reading. On the one hand, under everyday reading conditions, the automatic (in this sense, outside the conscious control of the reader) involvement of top-down influences can augment the visual input from a printed page and enhance the efficiency and accuracy with which visual information is used to read (see discussions by [45–47]). On the other hand, however, when inspecting images of the Shroud for writing, it is likely that the same automatic resources can lead to illusory perceptions of inscriptions based on visible content that has no links at all to the activity of writing. But there is no reason to suppose that individuals on both sides of the inscription debate would not be susceptible to the influence of these normal processes. Consequently, it remains to be determined why some observers of the Shroud are so convinced that inscriptions exist while others are similarly convinced that no inscriptions are present.

### A Role for Perceptual Expectations?

A possible source of this discrepancy is that human observers with opposing predispositions on the provenance of the Shroud have different expectations about the presence or absence of writing on its fabric, and these expectations lead to different perceptual experiences. More specifically, observers who believe in the religious provenance of the Shroud may be predisposed to perceive inscriptions in accord with these expectations, while those who do not believe in the religious provenance of the Shroud may be predisposed not to perceive such inscriptions. This is not to say that predispositions by either group produce conscious or deliberately misleading influences on judgments concerning the existence of writing. Indeed, there is good evidence that perceptual expectations (often described as perceptual set and response bias; see [48, 49]) created by different predispositions can alter greatly an individual’s detection of written language, and these effects can occur in a number of ways. For example, in an early classic study, Goldiamond and Hawkins [50] led participants to anticipate that some novel “words” (arbitrary sequences of letters) were more likely than others to appear in an experiment in which brief presentations were used to make stimuli difficult to see. In fact, in the experiment, all presentations were blank and no stimuli were actually presented, and yet the frequency with which participants reported seeing each “word” was influenced strongly by the expectations that had been generated before the experiment. Clearly, this effect could not be attributed to the accurate perception of sensory information since nothing relevant could have been seen, and the findings serve well to illustrate the substantial effects that expectations can produce when observers attempt to detect non-existent words in an image. In another classic study, influences exerted by the expected category to which words belonged were investigated. Reid, Henneman, and Long [51] presented participants with words that were visually degraded (blurred) so as to be barely legible. When presented without giving participants any expectations concerning the nature of these words, accurate word identification occurred on only 2% of occasions. However, when participants were informed that words were relevant to either, in one condition, baseball or, in another condition, American football, accurate identification increased substantially for words in each case. Moreover, this effect was larger when the categorical restriction was greater, and occurred both when category information was given before and after words were displayed. It seems, therefore, that when the physical content of words is barely legible [51] and even when words are non-existent [50], knowledge of the context in which stimuli are encountered can influence greatly how visual information is perceived and reported.

Other findings also suggest that the context in which words are encountered can affect word identification, and that these effects can be automatic, and greater when words are indistinct. In several well-established laboratory paradigms, when a target word is presented in a related semantic context (e.g., target word DOG preceded by CAT), responses to the target word show that this word is perceived more readily than in an unrelated semantic context (e.g., DOG preceded by BUS; for reviews, see [48, 49]). These findings are supported by studies indicating that a priming effect does not necessarily require conscious access to the prime or the target word [52–55] and that semantic context is especially influential when target words are visually indistinct (e.g., [56–60]). Indeed, other evidence [61] indicates that context can even lead to the illusory perception of words that are not actually present in a display. Consequently, when considering the perception of writing, it seems clear that the context in which a visual stimulus is encountered can produce crucial discrepancies between physical stimulation and the perceptual state of an observer.

The influences of context on word recognition reported so far in the literature are highly relevant to understanding the effects of observers’ expectations on the detection of inscriptions on the Shroud. In particular, since researchers examining the Shroud for inscriptions will naturally be aware of the Shroud’s religious significance and potential provenance, this context may be beneficial for detecting low-contrast inscriptions because the context provided by the Shroud itself may enhance an observer’s ability to detect and identify genuine inscriptions. However, and as we have seen from previous research, knowledge of the context in which stimuli are encountered can often inspire illusory perceptions. Consequently, in the case of the Shroud, belief in the religious provenance of the artifact may inspire observers to see inscriptions that are consistent with this belief but which are not actually present. Under conditions in which the existence of writing on an artifact cannot be certain, therefore, the detection of inscriptions may be driven inappropriately by the expectations of observers.

### An Investigation of the Influence of Religious Context on the Detection of Writing on a Linen Artifact

To date, no published empirical research has investigated the effects of religious context on the detection of writing on a linen artifact. Accordingly, the present research was conducted to cast light directly on the possibility that the context in which a linen artifact is viewed may affect the likelihood of religious writing being detected even when the absence of writing is ensured. To do this, we used a scanned image of a piece of innocuous modern linen with no religious provenance and which contained no writing, and this image was shown in two viewing contexts. In one context, participants were told that the image was of a linen artifact that was important to the Christian faith whereas participants in the other context were told that the image was of a simple piece of linen. If the context provided by the apparent religious provenance of an image leads to an increased predisposition to detect writing reflecting this provenance even when no writing is actually present, this effect should be revealed by a greater detection of religious words in the religious context condition. In contrast, if the apparent religious provenance of an image has no such effect, word detection performance should be similar for the religious and non-religious context conditions.

## Experiment 1

### Method

#### Ethics Statement

This research was conducted with the ethical approval of the Research Ethics Committee at Zayed University, and in accordance with the principles expressed in the Declaration of Helsinki. All participants gave informed consent in writing.

#### Participants

Participants were 32 university students (24 female, 8 male), fluent in English, aged between 18 and 21 years, and with normal or corrected-to-normal vision. All participants were naïve to the purpose of the experiment and volunteered to take part.

#### Design and Materials

**Fig 1:**
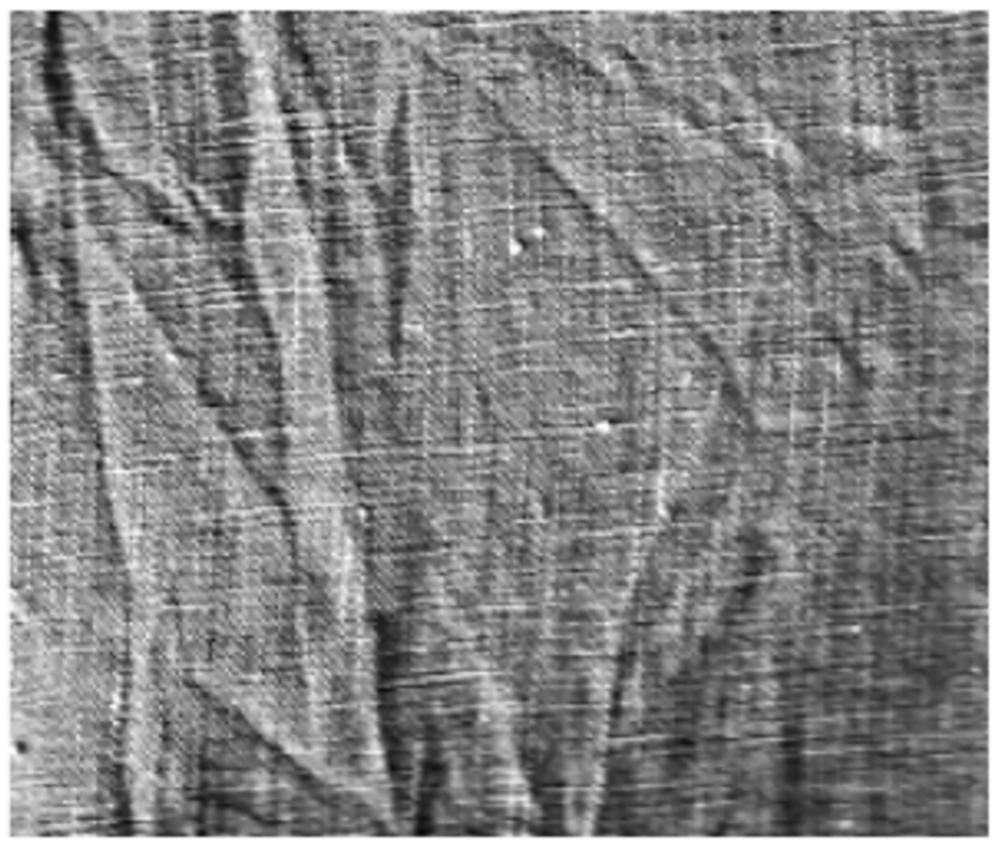
The image used in this study. The item scanned was a piece of modern linen, manufactured c.2009.

A section of modern linen (manufactured c.2009) was scanned using Photoshop software and printed so as to almost fill an A4 sheet of paper [see Fig 1]. The deliberate aim was not to replicate images of the actual Shroud since this procedure may have inspired unwanted biases in the perceived provenance of the fabric shown in the image. Instead, the aim was to present participants with an image with no inherent significant provenance and which was certain to contain no writing but which was well suited to revealing effects of context on the perception of writing under the types of conditions appropriate to studying the Shroud. The same image was shown to all participants and participants were assigned randomly to one of two context groups, with the constraint that each group contained 16 participants (12 female, 4 male). Participants in the Religious Context group were informed that the image was of an artifact that was important to the Christian faith, while participants in the non-religious Neutral Context group were informed that the image was of a simple piece of linen. Both groups were told that the image may contain faint words (in English) and were asked to report any words (or parts of words) they could see. All instructions were given in a printed format.

#### Procedure

Each participant took part individually and, after being given the appropriate instructions, inspected the image and wrote down any words that they thought they could see in the image. No time limitations were placed on responding. After listing the words they had detected, each participant was then required to trace the outline of each of these words on the image.

### Results

All response words were categorized as religious or non-religious for each context group (for totals and means, see Table 1). Each word was categorized by 10 independent judges and all words were determined unanimously by the judges as either religious or non-religious, and these categorizations were assessed further using the dictionary of the Association of Religion Data Archives (www.TheARDA.com). Religious words included God, Jesus, bible, church, and blessing, while non-religious words included cat, home, walk, button, and table. All participants were able to trace the outline of each of the words they reported and indicated that each word was visible in the image, albeit faint.

**Table 1:** Totals and means of religious and non-religious words detected in each context condition in Experiment 1. SD = Standard Deviation.

	Religious Context	Religious Context	Neutral Context	Neutral Context
	Religious Words	Non-Religious Words	Religious Words	Non-Religious Words
Total	27	8	0	6
Mean	1.7	0.5	0	0.4
SD	1.7	1.0	0	0.5

Mann-Whitney tests for the between-group data showed that more words were detected overall in the religious context condition than in the neutral context condition (35 vs 6; U = 39.0, z = 3.51, *p* < .001, *r* = .62). However, it should be clear from Table 1 that the effect of context was actually observed for the detection of religious words only and this was confirmed by further analyses. Specifically, context had no effect on the number of non-religious words detected (8 vs 6; U = 121.0, z = .32, *p* = .75, *r* = .06) but many more religious words were detected for the religious context than for the neutral context (27 vs 0; U = 24.0, z = 4.43, *p* < .001, *r* = .78). In a similar vein, Wilcoxon tests for the within-group data showed that, for the religious context, more detected words were religious than non-religious (27 vs. 8; W = 7, z = 2.54, *p* = .01, *r* = .45) while, for the neutral context, fewer detected words were religious than non-religious (0 vs. 6; W = 0, z = 2.45, *p* = .01, r = .43).

## Experiment 2

Experiment 1 was conducted to determine how the detection of words in an image of a woven artifact was influenced by the context in which the image was viewed. Although the influence of context was clear in the results, we wished to be certain that the study had sufficient power to reveal effects of context reliably (e.g. [62, 63]). In view of the novelty of this research, previous research could not provide an accurate indication of the sample size required. We therefore conducted a power analysis using G*Power [64] and the effect size obtained in Experiment 1 for the comparison between the number of religious words produced in religious and neutral contexts (the key comparison) in order to estimate the required sample size. Based on this effect size (r = .78), the analysis indicated that at least 37 participants per group would be required (with a power of .8) to detect an effect. Accordingly, to establish whether the same pattern of effects could be replicated in a study that had greater statistical power, Experiment 2 repeated Experiment 1 exactly with the exception that 80 participants were assigned randomly to the two context groups, with the constraint that each group contained 40 participants (28 female, 12 male). All participants were from the same population as in Experiment 1 and no participant had taken part in the previous experiment. As before, all participants were able to trace the outline of each of the words they reported and indicated that each word was visible, albeit faint.

Data were categorized and analyzed in the same way as in Experiment 1 (for totals and means, see Table 2). Religious words included God, holy, Jesus, bible, church, and blessing, while non-religious words included house, talk, sing, cat, button, and floor. Mann-Whitney tests for the between-group data showed that more words were detected overall in the religious context condition than in the neutral context condition (118 vs 23; U = 60.50, z = 5.89, *p* < .001, *r* = .76). However, it was again clear (see Table 2) that the effect of context was restricted to the detection of religious words and this was confirmed by further analyses. Mann-Whitney tests showed that context had no effect on the number of non-religious words detected (26 vs 23; U = 760.0, z = .39, *p* = .70, *r* = .04) but many more religious words were detected for the religious context than for the neutral context (92 vs 0; U = 0.0, z = 7.70, *p* < .001, *r* = .86). In addition, Wilcoxon tests showed that, for the religious context, more detected words were religious than non-religious (92 vs. 26; W = 780.0, z = 5.56, *p* < .001, *r* = .62) while, for the neutral context, fewer detected words were religious than non-religious (0 vs. 23; W = 231.0, z = 4.41, *p* < .001, r = .49).

**Table 2:** Totals and means of religious and non-religious words detected in each context condition in Experiment 2. SD = Standard Deviation.

	Religious Context	Religious Context	Neutral Context	Neutral Context
	Religious Words	Non-Religious Words	Religious Words	Non-Religious Words
Total	92	26	0	23
Mean	2.3	0.7	0	0.6
SD	0.7	0.7	0	0.6

## Discussion

The findings of this study provide an important and novel indication of how the detection of writing on an image of a woven artifact is influenced by the context in which the image is viewed. In particular, although participants in both experiments viewed exactly the same image in each context condition, more religious words were detected in the Religious Context condition than in the Neutral Context condition, whereas the two contexts showed no effect on the number of non-religious words detected. Moreover, in the Neutral Context condition, no religious words at all were detected in either experiment.

These findings provide a clear indication that effects of religious context/expectations have a rather specific outcome on the detection of illusory writing. Indeed, it is worth emphasizing that it would be misguided to interpret the effects produced by context in this study as influences on the *total* number of words detected since changes in context affected the detection of religious words *only*. In addition, it should also be clear that religious and non-religious neutral contexts were used in these experiments because of the relevance of these contexts to investigating the expectations that specifically surround the detection of inscriptions on the Shroud. Other types of context would not have been suitable for this particular purpose but future research may choose to observe the influence of other appropriate contexts when considering the reports of writing on other artifacts with different types of provenance, either actual or assumed. For example, it remains to be seen if reports of indistinct writing on artifacts such as documents, weapons, pottery, metal vessels, and jewelry (e.g., [65, 66]) are also affected by the expectations of observers. In these situations, expectations may be driven by provenances based on such things as an artifact’s age, where it was found, its use, and the culture to which it belonged, and illusory perceptions of writing may then occur in line with these expectations.

The influences of context observed in this study are broadly consistent with the findings from previous research concerning the more general effects of context on language perception [see Introduction] but now contextualize these effects specifically in a situation related more closely to that which exists when viewing images of the Shroud. In particular, previous research suggests that increased expectations produced by the context in which an image is viewed may increase sensitivity to the perception of faint, indistinct images, and images of this kind clearly lie at the heart of claims that inscriptions exist on the Shroud. Consequently, these effects of context on perception could be highly beneficial when viewing the Shroud but this benefit can occur only if the visual content of the image reflects the actual existence of writing. In contrast, the findings of our study indicate that expectations generated by religious provenance increase substantially the chances of perceiving religious writing in an image where no writing is actually present, and so, rather than being beneficial, it seems that such expectations increase substantially the likelihood of perceiving illusory inscriptions; that is, perceiving inscriptions that do not actually exist.

Importantly, the performance of the participants in our study indicates that the reports of writing obtained reflect actual perceptions rather than report bias. In particular, participants indicated where in the image they had seen a particular word and were able to trace the outlines of all the words that they detected. Thus, although these perceptions were clearly influenced considerably by expectations produced by the context in which the image was viewed, and although the words perceived were reported as faint, this aspect of the experiment indicates that context affected the perceptual state of participants and did not merely inspire a bias towards reporting the existence of words with no perceptual basis (cf the study by Goldiamond & Hawkins [50]).

It is also useful to note that while our study has shown that religious context inspires the illusory perception of religious writing in an image of innocuous woven material, and that the observers who took part produced a total of no less than 119 detections of religious words when informed that the artifact had religious significance, it is likely that the overall size of the Shroud, and the religious fervor surrounding its existence and provenance, inspire an even greater number of illusory perceptions on the artifact itself. In particular, the image used in our study was of a small piece of linen whereas images of the Shroud cover a far greater area (the Shroud is approximately 4.4 m long and 1.1 m wide). In addition, Brugger and Graves [67], and Riekki, Lindeman, Aleneff, Halme and Nuortimo [68] suggest that observers who are more religious are more likely than non-religious observers to see meaningful patterns in ambiguous stimuli. Thus, observers attracted to the topic of the Shroud because of their religious beliefs may be extraordinarily predisposed to perceive illusory religious inscriptions (see also [69]) and, as a consequence, be likely to report the detection of many more religious words on the Shroud than were observed in our study.

In sum, the aim of this research has not been to question the authenticity of the Shroud or the presence of images of a human body and face. Instead, our focus has been the claims made concerning the existence of religious inscriptions which many believe cast crucial light on the provenance of this important artifact. Accordingly, we have considered a range of psychological processes that are likely to be involved in the detection and perception of inscriptions on the Shroud. In particular, we report previous research that has shown that what people perceive is often rather different from the sensory information producing these perceptions, and that context can lead easily to perceptions of illusory letters and illusory words that are not actually present in a display. This influence of context was then demonstrated empirically for religious expectations under conditions designed to be closer to those surrounding the detection of writing on the Shroud. As a result, it seems reasonable to conclude that the context in which a linen artifact is viewed can produce important discrepancies between sensory stimulation and the perceptual state of an observer, and there may be few situations where these effects are more influential than when determining the existence of inscriptions on such a famous artifact as the Shroud of Turin.

## Supporting Information

S1 Data (PDF)

S2 Data (PDF)
