# Supplementary material for: Seeing Inscriptions on the Shroud of Turin: The Role of Psychological Influences in the Perception of Writing
Source: PLoS One. 2015 Oct 28;10(10):e0136860. doi: 10.1371/journal.pone.0136860 (PMC4624961; doi:10.1371/journal.pone.0136860)
Supplement: S1 Data — (PDF) [file pone.0136860.s001.pdf]

	Condition 1<br>Religious Context<br>Words Reported		Condition 2<br>Neutral Context<br>Words Reported	
Participant	**Religious**	**Non-Religious**	**Religious**	**Non-Religious**
1	1 (God)	0	0	0
2	0	0	0	0
3	2 (Jesus, Bible)	0	0	1 (Reflex)
4	2 (Jesus, Cross)	2 (Walk, Cat)	0	1 (Table)
5	0	0	0	1 (Paper)
6	0	0	0	1 (Leg)
7	2 (Holy, Temple)	0	0	0
8	3 (Jesus, Christ, Crucifix)	2 (Sing, Button)	0	0
9	1 (Prayer)	3 (Home, Duck, To)	0	0
10	1 (Christ)	0	0	0
11	2 (Blessing, God)	0	0	0
12	3 (Church, Jesus, Christ)	0	0	0
13	2 (Demon, Holy)	0	0	0
14	3 (Jesus, God, Sin)	0	0	0
15	2 (Bible, Hell)	1 (Table)	0	1 (Bus)
16	3 (Nazareth, God, Jew)	0	0	1 (Walk)
**Totals**	**27**	**8**	**0**	**6**
