# Supplementary material for: Seeing Inscriptions on the Shroud of Turin: The Role of Psychological Influences in the Perception of Writing
Source: PLoS One. 2015 Oct 28;10(10):e0136860. doi: 10.1371/journal.pone.0136860 (PMC4624961; doi:10.1371/journal.pone.0136860)
Supplement: S2 Data — (PDF) [file pone.0136860.s002.pdf]

	Condition 1<br>Religious Context<br>Words Reported		Condition 2<br>Neutral Context<br>Words Reported	
Participant	Religious	Non-Religious	Religious	Non-Religious
1	2 (Holy, Church)	0	0	0
2	3 (God, Blessing, Cross)	1 (Cat)	0	0
3	2 (Bible, Belief)	0	0	1 (Leg)
4	3 (Jesus, Temple, Nazareth)	1 (Leg)	0	0
5	2 (Crucifix, Prayer)	0	0	1 (Mess)
6	3 (Blessing, Altar, Jesus)	0	0	1 (Hold)
7	2 (Jesus, God)	0	0	0
8	3 (Lord, Jesus, Christ)	2 (Talk, Table)	0	0
9	3 (Jesus, Church, Satan)	1 (Walk)	0	0
10	1 (Christ)	0	0	0
11	2 (Spirit, Jesus)	0	0	1 (Walk)
12	3 (Divine, God, Jesus)	1 (Long)	0	0
13	2 (Crucifix, Prophet)	0	0	0
14	3 (God, Church, Bible)	0	0	0
15	2 (Holy, Jew)	1 (Paper)	0	1 (Care)
16	3 (Temple, Jesus, Christ)	0	0	1 (Seen)
17	2 (Satan, Holy)	1 (Floor)	0	0
18	3 (God, Temple, Cross)	1 (Button)	0	1 (Button)
19	2 (Jesus, Crucifix)	0	0	0
20	3 (Nazareth, God, Altar)	1 (Mess)	0	0
21	2 (Jesus, Bible)	1 (Reflex)	0	0
22	1 (God)	0	0	1 (Sing)
23	2 (Bible, Church)	1 (Care)	0	1 (To)

24	1 (Jesus)	0	0	2 (Pride, Unit)
25	3 (Bible, Blessing, God)	1 (Bus)	0	1 (Bean)
26	4 (Sacred, Holy, Divine, God)	0	0	1 (House)
27	2 (Jesus, Holy)	1 (Sing)	0	2 (My, Older)
28	3 (Bible, Church, God)	0	0	0
29	2 (Belief, Jesus)	1 (Drive)	0	1 (State)
30	2 (God, Christ)	0	0	1 (Mist)
31	3 (Holy, Temple, Jew)	1 (Hold)	0	1 (Hat)
32	2 (God, Church)	1 (Bus)	0	1 (No)
33	1 (Jesus)	0	0	0
34	2 (Blessing, God)	1 (Walk)	0	1 (Duck)
35	3 (Nazareth, Church, Holy)	2 (Home, Week)	0	0
36	2 (Jesus, God)	1 (See)	0	1 (Six)
37	1 (Christian)	0	0	1 (Handle)
38	2 (Christ, Jesus)	2 (Bean, Deep))	0	0
39	3 (Commandment, God, Holy)	2 (Hold, Drive)	0	1 (Bold)
40	2 (God, Jesus)	1 (House)	0	0
**Totals**	**92**	**26**	**0**	**23**
